# Activating Mutations of the G-protein Subunit α _11_ Interdomain Interface Cause Autosomal Dominant Hypocalcemia Type 2

**DOI:** 10.1210/clinem/dgz251

**Published:** 2019-12-10

**Authors:** Caroline M Gorvin, Victoria J Stokes, Hannah Boon, Treena Cranston, Anna K Glück, Shailini Bahl, Tessa Homfray, Theingi Aung, Brian Shine, Kate E Lines, Fadil M Hannan, Rajesh V Thakker

**Affiliations:** 1 Academic Endocrine Unit, Radcliffe Department of Medicine, Oxford Centre for Diabetes, Endocrinology and Metabolism (OCDEM), University of Oxford, Oxford, UK; 2 Oxford NIHR Biomedical Research Centre, University of Oxford, Churchill Hospital, Oxford, UK; 3 Oxford Molecular Genetics Laboratory, Churchill Hospital, Oxford, UK; 4 Department of Paediatrics, Ashford and St. Peter’s Hospitals NHS Foundation Trust, Surrey, UK; 5 Department of Clinical Genetics, St George’s University Hospital, London, UK; 6 The Centre for Diabetes and Endocrinology, Royal Berkshire NHS Foundation Trust, Reading, UK; 7 Department of Clinical Biochemistry, John Radcliffe Hospital, Oxford University Hospitals NHS Trust, Oxford, UK

**Keywords:** G-protein, calcium-sensing receptor, parathyroid hormone

## Abstract

**Context:**

Autosomal dominant hypocalcemia types 1 and 2 (ADH1 and ADH2) are caused by germline gain-of-function mutations of the calcium-sensing receptor (CaSR) and its signaling partner, the G-protein subunit α _11_ (Gα _11_), respectively. More than 70 different gain-of-function CaSR mutations, but only 6 different gain-of-function Gα _11_ mutations are reported to date.

**Methods:**

We ascertained 2 additional ADH families and investigated them for CaSR and Gα _11_ mutations. The effects of identified variants on CaSR signaling were evaluated by transiently transfecting wild-type (WT) and variant expression constructs into HEK293 cells stably expressing CaSR (HEK-CaSR), and measuring intracellular calcium (Ca^2+^_i_) and MAPK responses following stimulation with extracellular calcium (Ca^2+^_e_).

**Results:**

CaSR variants were not found, but 2 novel heterozygous germline Gα _11_ variants, p.Gly66Ser and p.Arg149His, were identified. Homology modeling of these revealed that the Gly66 and Arg149 residues are located at the interface between the Gα _11_ helical and GTPase domains, which is involved in guanine nucleotide binding, and this is the site of 3 other reported ADH2 mutations. The Ca^2+^_i_ and MAPK responses of cells expressing the variant Ser66 or His149 Gα _11_ proteins were similar to WT cells at low Ca^2+^_e_, but significantly increased in a dose-dependent manner following Ca^2+^_e_ stimulation, thereby indicating that the p.Gly66Ser and p.Arg149His variants represent pathogenic gain-of-function Gα _11_ mutations. Treatment of Ser66- and His149-Gα _11_ expressing cells with the CaSR negative allosteric modulator NPS 2143 normalized Ca^2+^_i_ and MAPK responses.

**Conclusion:**

Two novel ADH2-causing mutations that highlight the Gα _11_ interdomain interface as a hotspot for gain-of-function Gα _11_ mutations have been identified.

Autosomal dominant hypocalcemia (ADH) is a disorder of systemic calcium homeostasis, which affects the parathyroid glands and kidneys, and is caused by increased sensitivity of the calcium-sensing receptor (CaSR) to extracellular calcium (Ca^2+^_e_) concentrations (1, 2). The CaSR is a class C G-protein coupled receptor (GPCR), which plays a pivotal role in the parathyroid and renal regulation of Ca^2+^_e_ (3) by activating the G-protein α _q/11_ family, which enhances phospholipase C (PLC) activity resulting in intracellular calcium (Ca^2+^_i_) mobilization and activation of the phospho-ERK (pERK) arm of the MAPK signaling pathway (3, 4) (Fig. 1A). ADH comprises 2 genetic variants, designated as ADH types 1 and 2 (ADH1 and ADH2), which are caused by germline gain-of-function mutations in genes encoding the CaSR and G-protein subunit α _11_ (Gα _11_) proteins, respectively (1, 2). ADH1 (OMIM #601198) has been reported in association with >70 different CaSR mutations (5), and is characterized by hypocalcemia, hyperphosphatemia, hypomagnesemia, inappropriately low or normal PTH concentrations, and a relative or absolute hypercalciuria (1, 6). In addition, some ADH1 patients may develop a Bartter-like syndrome with hypokalemic alkalosis, renal salt wasting, and hyperreninemic hyperaldosteronism (7, 8). ADH2 (OMIM #615361) has been described in 7 probands (2, 9–12), and these patients have a similar serum biochemical phenotype to that of ADH1 patients. However, ADH2 is associated with a milder urinary phenotype, with significantly reduced urinary calcium excretion compared with ADH1 (9). Moreover, short stature has been reported in 2 ADH2 kindreds (9, 12). Conversely, germline loss-of-function CaSR and Gα _11_ mutations lead to the opposite phenotypes of familial hypocalciuric hypercalcemia (FHH) types 1 and 2, respectively (1, 2), which is characterized by lifelong elevations of serum calcium concentrations in association with normal or mildly raised serum PTH concentrations, and low urinary calcium excretion (calcium‐to‐creatinine clearance ratio < 0.01) (13, 14).

**Figure 1.**
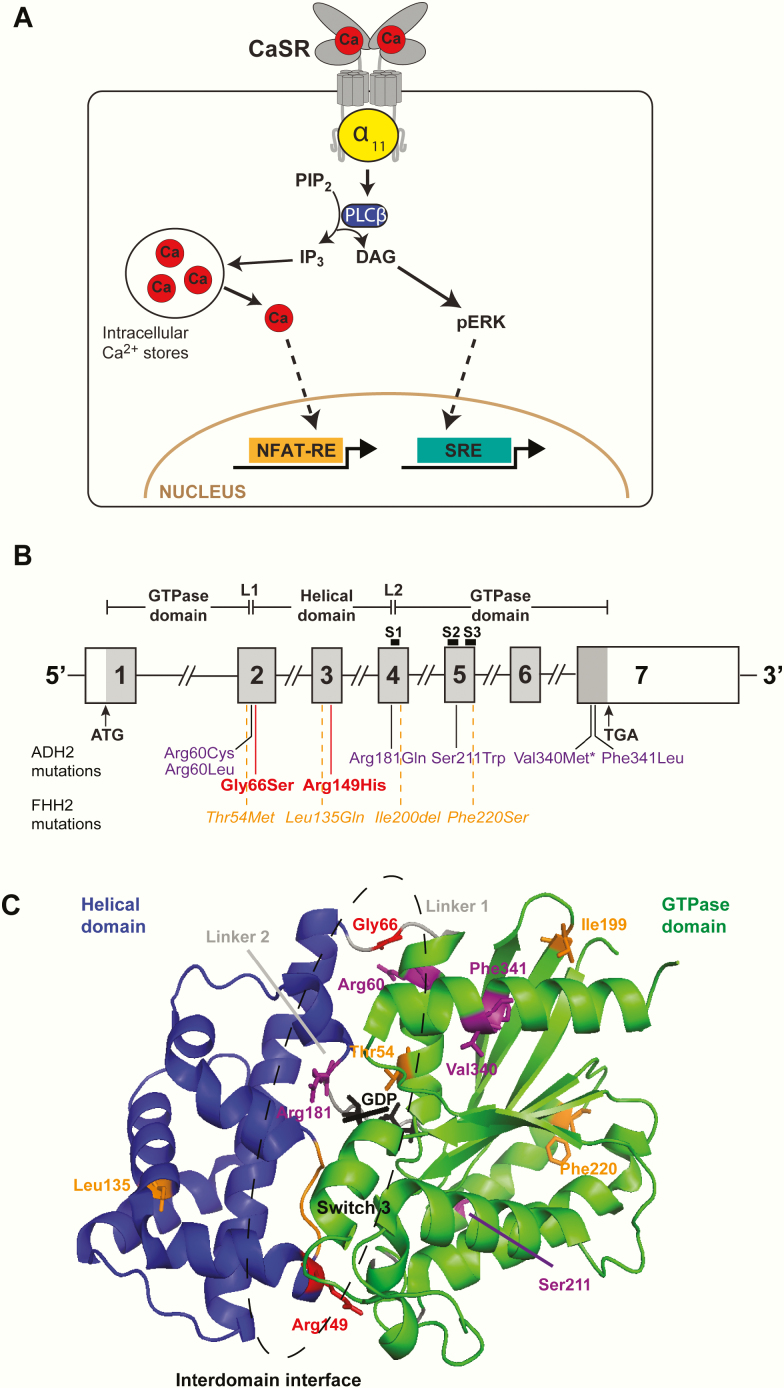
(A) Diagrammatic representation of the CaSR signaling pathways. Calcium (red circle, Ca) binding to CaSR (gray) activates Gα _11_ (yellow)-dependent stimulation of phospholipase C-β (PLCβ) (dark blue), which catalyzes formation of inositol 1,4,5-trisphosphate (IP_3_) and diacylglycerol (DAG) from phosphatidylinositol 4,5-bisphosphate (PIP_2_). IP_3_ mediates calcium mobilization from intracellular stores, whereas DAG activates the pERK signaling cascade. Gene transcription mediated by intracellular calcium and pERK can be measured using nuclear factor of activated T-cell response element (NFAT-RE) and serum response element (SRE) containing luciferase reporter constructs, respectively. (B) Schematic representation of the genomic organization of the human *GNA11* gene that comprises 7 exons, with coding regions shaded gray and untranslated regions represented by open boxes. Loss-of-function Gα _11_ mutations (orange), have been reported in 4 FHH2 probands (2, 15, 16), and 6 gain-of-function Gα _11_ mutations (purple), reported in 7 ADH2 probands (2, 9–12). The Val340Met Gα _11_ mutation (asterisk) has been reported in 2 unrelated ADH2 probands (11, 12). This manuscript describes 2 ADH2 mutations, p.Gly66Ser and p.Arg149His (red). The GTPase domain (encoded by portions of exon 1, 2, and 4, and the whole of exons 5–7) is connected to the helical domain (encoded by portions of exon 2 and 4, and the whole of exon 3) by the linker 1 (L1) and linker 2 (L2) peptides. Three flexible switch regions (S1-S3), which undergo conformational changes upon Gα _11_ activation, are encoded by exons 4 and 5. (C) Homology model of the Gα _11_ protein based on the structure of Gα _q_ in complex with PLCβ (PDB: 3OHM) (26). The Gα helical (blue) and GTPase (green) domains are connected by the linker 1 and linker 2 peptides (gray). GDP (black) is bound at the interdomain interface (dashed ellipse). Switch 3 is shown in orange, and the Gly66 and Arg149 Gα _11_ residues that are mutated in families 1 and 2 (Fig. 2), respectively, shown in red. Residues previously reported to harbor 6 ADH2 and 4 FHH2 Gα _11_ mutations are shown in purple and orange, respectively (2, 9–12, 15, 16). Adapted from Hannan FM et al J Mol Endocrinol 2016 Oct;57 (28):R127-42.

To date, 4 FHH2 and 6 ADH2 different mutations have been identified in the *GNA11* gene on chromosome 19p13.3 (Fig. 1B), which encodes Gα _11_, and studies of the location of such mutations has provided insight into Gα _11_ structure function (2, 9–11, 15, 16). Thus, FHH2 and ADH2 mutations cluster within 3 regions (Fig. 1C): the Gα _11_-GPCR interaction region; the interdomain interface between the helical and GTPase domains; and the sites at which Gα _11_ interacts with Gβγ and PLC (2, 9–11, 15, 16). This indicates that these 3 structural regions play a critical role in Gα _11_-mediated CaSR signaling. Additionally, previous studies of these mutations have indicated that CaSR negative allosteric modulators, which are known as calcilytic compounds, can normalize the gain-of-function caused by Gα _11_ mutations both *in vitro* and in mouse models of ADH2 (17–19), and thus represent a potential targeted therapy for this disorder.

Here, we report the clinical and genetic findings in 2 unrelated families with ADH, in whom novel heterozygous germline gain-of-function Gα _11_ mutations, were identified.

## Materials and Methods

### Patients and families

#### Family 1.

**Figure 2.**
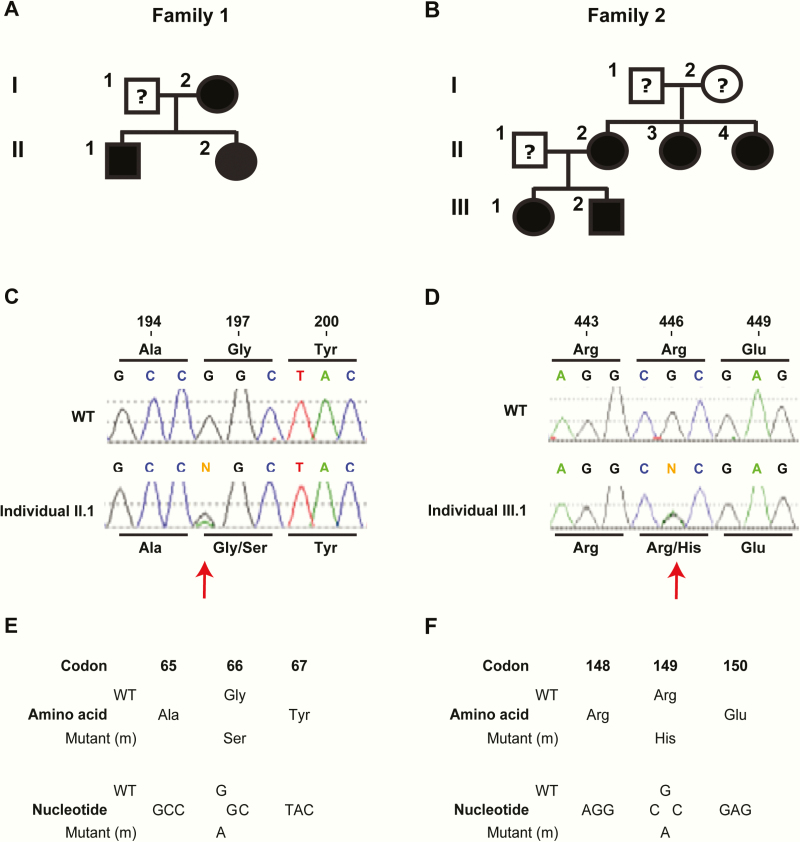
Identification of ADH2 mutations, p.Gly66Ser and p.Arg149His, in Gα _11_. Pedigrees of (A) family 1 and (B) family 2, with males and females indicated by squares and circles, respectively. Individuals affected with hypocalcemia are indicated by filled symbols; and the spouses of individuals I.2 (family 1) and II.2 (family 2), and parents of individuals II.2, II.3 and II.4 (family 2) who were not available and therefore their affected status remained unknown are shown as open symbols with a question mark. (C) DNA sequence analysis showed the hypocalcemic patients (individual II.1 shown) from family 1 to be heterozygous for a G-to-A transition at c.196 (red arrow) within exon 2 of *GNA11*. (D) DNA sequence analyses of the hypocalcemic patients (individual III.1 shown) from family 2 showed them to be heterozygous for a G-to-A transition at c.446 (red arrow) within exon 3 of *GNA11*. (E) The G-to-A transition in family 1 was predicted to lead to a missense substitution of Gly to Ser at codon 66. (F) The G-to-A transition in family 2 was predicted to lead to a missense substitution of Arg to His at codon 149.

This family comprised 3 affected members (a mother, her son, and daughter) (Fig. 2A). The son (individual II.1, Fig. 2A) at the age of 10 years was referred with a chronic motor tic disorder, which was subsequently diagnosed as Tourette syndrome. He was also experiencing paresthesia, and biochemical investigations showed him to have a mildly low serum calcium of 2.12 mmol/L (normal 2.20–2.70 mmol/L) in association with an inappropriately normal plasma PTH of 2.8 pmol/L (normal 1.0–7.0 pmol/L) and insufficient serum 25-hydroxyvitamin D of 42 nmol/L (adequate >50 nmol/L). He had a normal serum phosphate concentration of 1.57 mmol/L (normal 0.90–1.80), magnesium of 0.90 mmol/L (normal 0.70–1.0), creatinine of 59 μmol/L (normal 28–63), alkaline phosphatase activity of 146 IU/L (normal 60–425), and low urinary calcium-to-creatinine ratio of 0.08 mmol/mmol (normal 0.30–0.70). He was commenced on oral calcium and cholecalciferol, which increased his serum 25-hydroxyvitamin D to 87 nmol/L; however, his serum calcium remained low at 2.11 mmol/L. His mother and younger sister (Fig. 2A) were also found to be mildly hypocalcemic with serum calcium concentrations of 2.08 mmol/L and 2.15 mmol/L, respectively. This family was investigated for ADH as a possible cause of the mild hypocalcemia and leukocyte DNA was obtained from affected family members following informed consent for analysis of the *CASR* and *GNA11* genes.

#### Family 2.

This family comprised 5 affected members (a mother and her 2 sisters, and her daughter and son) (Fig. 2B). The daughter (individual III.1, Fig. 2B), at the age of 38 years, was referred with a 6-month history of fatigue, myalgia, dizziness, and bilateral hip pain. She had no history of paresthesia, muscle cramps, seizures, or renal calculi. She had not previously undergone neck surgery and had no history of deafness, renal or cardiac abnormalities, candidiasis, or Addison’s disease. Her only comorbidity was recently diagnosed autoimmune hypothyroidism, which was treated with levothyroxine 75 μg daily. Her height was 170 cm and weight was 84.3 kg. Biochemical investigations showed her to have a low serum calcium of 1.97 mmol/L (normal 2.20–2.60) in association with an inappropriately normal plasma PTH of 4.1 pmol/L (normal 1.0–7.0), and a borderline low urine calcium to creatinine ratio of 0.30 mmol/mmol (normal 0.30–0.70), and a fractional excretion of calcium 0.01 (normal >0.01). She had a normal serum magnesium concentration of 0.78 mmol/L (normal 0.70–1.00), phosphate of 1.12 mmol/L (normal 0.70–1.45), creatinine of 69 μmol/L (normal 45–90), alkaline phosphatase activity of 45 U/L (normal 30–130), 25-hydroxyvitamin D of 125 nmol/L (normal>50 nmol/L), 1,25-dihydroxyvitamin D of 111 pmol/L (normal 43–144), and TSH of 1.76 mU/L (normal 0.30–4.20). Thyroid peroxidase antibodies were elevated at >1518 (normal < 60 IU/mL), and anti-parathyroid antibodies, as assessed by indirect immunofluorescence (20), were not detected. Her hypocalcemia was initially treated with 1.0 to 2.5 g of oral elemental calcium daily. However, she remained hypocalcemic and also became hypomagnesemic (lowest serum magnesium = 0.62 mmol/L), and was commenced on alfacalcidol 1.0 μg daily, as well as oral magnesium aspartate 10 mmol twice daily. Her mother, brother, and 2 maternal aunts were also hypocalcemic (Fig. 2B), and these findings were suggestive of either ADH or familial isolated hypoparathyroidism. Leukocyte DNA was obtained from affected family members following informed consent for analysis of the *CASR, GNA11, GCM2, GATA3, AIRE*, and *PTH* genes.

### Mutational analysis

Mutational analysis was performed according to the clinical indications using leukocyte DNA, and by Sanger sequencing of all coding exons and exon-intron boundaries, using exon-specific primers (Sigma Aldrich), the BigDye Terminator v3.1 Cycle Sequencing Kit (Life Technologies), and an automated detection system (ABI3730 Automated capillary sequencer; Applied Biosystems), as previously reported (2, 21). Investigation of potentially pathogenic variants was undertaken using the publicly accessible Genome Aggregation Database (gnomAD): (https://gnomad.broadinstitute.org/) which is a dataset comprising 125 748 exome sequences and 15 708 whole-genome sequences from unrelated individuals. Predicted effects of the mutations was assessed using Polyphen-2 (http://genetics.bwh.harvard.edu/pph2/) (22) and MutationTaster (http://www.mutationtaster.org/) (23).

### Protein sequence alignment and 3-dimensional modeling of Gα_11_ structure

Protein sequences of Gα _11_ orthologs and Gα paralogs were aligned using ClustalOmega (http://www.ebi.ac.uk/Tools/msa/clustalo/) (24). Gα _11_ 3-dimensional modeling was undertaken using the reported 3-dimensional structure of Gα _q,_ which shares 90% identity at the amino acid level with Gα _11_ (2). Gα _q_ was modeled in complex with the small molecule inhibitor YM-254890 (Protein Data Bank [PDB] accession no. 3AH8) (25) and also in complex with the phospholipase C β3 effector (PDB: 3OHM) (26). Molecular modeling was performed using The PyMOL Molecular Graphics System (Version1.2r3pre, Schrödinger, LL Pymol) (2).

### Cell culture and transfection

Wild-type (WT) and mutant pBI-CMV2-*GNA11* expression constructs were generated as described (2), and transiently transfected into HEK293 cells stably expressing CaSR (HEK-CaSR) (2) using Lipofectamine 2000 (Life Technologies). The bidirectional pBI-CMV2 cloning vector was used as it facilitated the coexpression of Gα _11_ and GFP (2), and site-directed mutagenesis was used to generate the mutant *GNA11* construct using the Quikchange Lightning Site-directed Mutagenesis kit (Agilent Technologies) and gene-specific primers (Sigma Aldrich), as described (16). Cells were maintained in DMEM-Glutamax media (Thermo Fisher) with 10% fetal bovine serum (Gibco) and 400 µg/mL geneticin (Thermo Fisher) at 37ºC, 5% CO_2_. Successful transfection was confirmed by visualizing GFP fluorescence using an Eclipse E400 fluorescence microscope with a Y-FL Epifluorescence attachment and a triband 4,6-diamidino-2-phenylindole-FITC-Rhodamine filter, and images captured using a DXM1200C digital camera and NIS Elements software (Nikon) (2, 16). The expression of Gα _11_ and CaSR proteins was also determined by Western blot analysis using anti-Gα _11_ (Santa-Cruz), anti-GFP (Santa Cruz), or anti-CaSR (Abcam) antibodies; calnexin expression was used as a loading control and detected using an anti-calnexin (Millipore) antibody. The Western blots were visualized using an Immuno-Star WesternC kit (BioRad) on a BioRad Chemidoc XRS + system (2).

### Intracellular calcium measurements

Ca^2+^_e_-induced Ca^2+^_i_ responses were measured by Fluo-4 calcium assays as previously described (16). HEK-CaSR cells were plated in 12-well plates and transiently transfected with 1000 ng/mL pBI-CMV2-*GNA11*. Following 24 hours’ incubation, cells were replated at 30 000 cells/well in black-walled 96-well plates (Corning). Cells were treated with serum-free media (SFM) overnight. Fluo-4 dye was prepared according to manufacturer’s instructions (Invitrogen), and cells loaded for 1 hour at 37°C. Baseline measurements were made and increasing concentrations of CaCl_2_ injected automatically into each well. Changes in Ca^2+^_i_ were recorded on a PHERAstar instrument (BMG Labtech) at 37°C with an excitation filter of 485 nm and an emission filter of 520 nm. The peak mean fluorescence ratio of the transient response after each individual stimulus was measured using Cytomation Summit software (Beckman Coulter), and expressed as a normalized response. Nonlinear regression of concentration-response curves was performed with GraphPad Prism using the normalized response at each [Ca^2+^]_e_ for each separate experiment for the determination of the mean half-maximal concentration (EC_50_) (i.e., [Ca^2+^]_e_ required for 50% of the maximal response). Assays were performed in 4 to 8 independent transfections. Statistical analysis was performed using the *F*-test.

### Luciferase reporter assays

HEK-CaSR cells were plated in 24-well plates and transiently transfected with 100 ng/mL pBI-CMV2-*GNA11* WT or mutant construct, 100 ng/mL luciferase construct (either pGL4-nuclear factor of activated T-cell response element [NFAT-RE] or pGL4-serum response element [SRE] and 10 ng/mL pRL null control luciferase reporter. Following 48 hours incubation, cells were treated with SFM overnight. Cells were then treated with SFM containing 0.1 to 10 mM CaCl_2_ and incubated for 4 hours. Cells were lysed and assays performed using Dual-Glo Luciferase (Promega) on a Veritas Luminometer (Promega) as previously described (16, 18). Luciferase:renilla ratios were expressed as fold changes relative to responses at low CaCl_2_ concentrations (0.1 mM). For studies with NPS 2143 (Abcam), drug was added to cells 4 hours before reporter assays were performed. All assay conditions were performed in 4 to 12 independent transfections. Statistical analysis was performed by 2-way ANOVA with Tukey’s multiple-comparisons test using GraphPad Prism 6.

## Results

### Identification of novel missense mutations in Gα_11_ in 2 ADH2 probands

**Figure 3.**
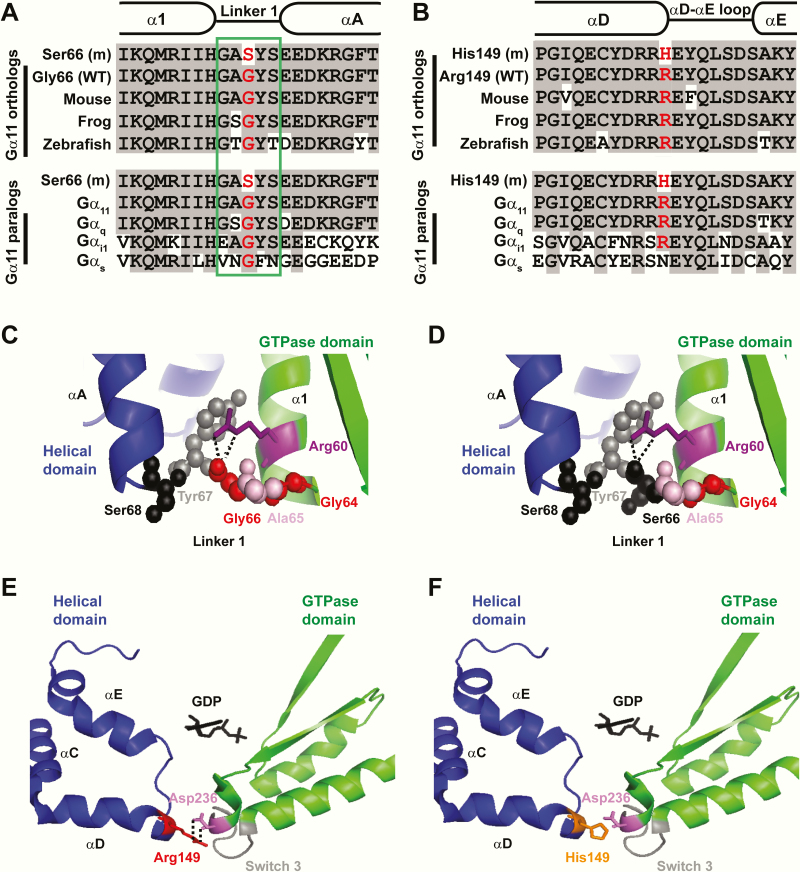
Predicted effects of the p.Gly66Ser and p.Arg149His mutations on the Gα _11_ protein by analysis of evolutionary conservation and structural modeling. (A-B) Multiple protein sequence alignment of Gα _11_-subunit orthologs (top) and Gα-subunit paralogs (bottom), with residues comprising the α1 helix, linker 1 peptide (green box) and αA helix shown in A; and residues of the αD helix, αD-αE loop, and αE helix shown in (B). Conserved residues are shown in gray, and WT and mutant (m) residue in red in panels A and B. Gly66 and Arg149 are highly conserved in Gα _11_ orthologs and Gα paralogs. (C-D) Homology model of the Gα _11_ protein based on the structure of Gα _q_ in complex with an inhibitor (PDB: 3AH8) (25). (C) The WT Gly66, which is a nonpolar hydrophobic amino acid, is located within linker 1 and forms a contact with Arg60 (purple), which is a positively charged hydrophilic amino acid. (D) Mutation of Gly66 to serine (Ser66), a hydrophilic amino acid leads to the introduction of a polar side chain, which projects into the cytoplasm and may alter the tight packing of the linker 1 region. The contact between Arg60 and Ser66 is unaffected. (E-F) Homology model of Gα _11_ based on the structure of Gα _q_ in complex with the phospholipase C β3 protein (PDB: 3OHM) (26). (E) The WT Arg149 is located within the αD helix of the helical domain, and forms polar contacts (hatched line) with the Asp236 residue in switch 3. (F) Mutation of Arg149 to His149, likely causes loss of the polar contact with Asp236, and may affect the interaction of the αD-helix with switch 3.

DNA sequence analyses in the 2 ADH families identified abnormalities only in the *GNA11* gene. Thus, in family 1 (Fig. 2A), a heterozygous G-to-A transition at nucleotide c.196 within exon 2 of *GNA11* (Fig. 1B and 2C) was identified and in family 2 (Fig. 2B), a heterozygous G-to-A transition at nucleotide c.446 within exon 3 of *GNA11* was identified (Fig. 1B and 2D). The G-to-A transition in family 1 is predicted to lead to a missense substitution of Gly to Ser at codon 66 of the Gα _11_ protein (Fig. 2E), and in family 2 to a missense substitution of Arg to His at codon 149 of the Gα _11_ protein (Fig. 2F). Bioinformatic analyses using Polyphen-2 and MutationTaster software (22, 23) predicted the p.Gly66Ser and p.Arg149His variants to be damaging and likely disease causing (Polyphen-2 score 1, MutationTaster score 0.99). The p.Arg149His Gα _11_ variant was not detected in the gnomAD database, whereas the p.Gly66Ser variant was detected in 2 of 281 488 alleles, yielding a rare allele frequency of <0.001%. The p.Gly66Ser and p.Arg149His variants were detected in all hypocalcemic members of families 1 and 2, respectively (Fig. 2A and 2B), and these findings with the demonstration of evolutionary conservation of the Gly66 and Arg149 residues in Gα _11_ orthologs and Gα paralogs (Fig. 3A and 3B), indicated that the p.Gly66Ser and p.Arg149His abnormalities likely represented pathogenic mutations rather than benign polymorphic variants. Thus, 2 heterozygous novel missense germline mutations (Fig. 2C–F) were likely identified in the 2 ADH families, and structural and functional characterization of these potential Gα _11_ mutations were therefore undertaken.

### Structural characterization of the p.Gly66Ser and p.Arg149His Gα_11_ mutant proteins

The Gly66 residue is located within the linker 1 peptide that acts as a flexible hinge between the helical and GTPase domains of Gα _11_ and connects the α1 helix of the GTPase domain with the αA helix of the helical domain (Figs. 1C, 3A, and 3C–D). The linker 1 peptide comprises 5 residues that form a hydrogen bond network with residues within the α1- and αA-helices to stabilize the G-protein structure (27) (Figs. 1C and 3C). The Gly66 residue represents the central amino acid of the linker 1 peptide and forms a hydrogen bond with the Arg60 residue (27), mutations of which have been reported to cause ADH2 (Figs. 1 and 3C) (9, 10). However, the Ser66 mutation is not predicted to disrupt the interaction with the Arg60 residue (Fig. 3D), but the mutant Ser66 residue instead leads to the introduction of a bulky polar side chain (Fig. 3D), which may destabilize the linker 1 region.

The Arg149 residue is located within the αD helix of the helical domain, which lies close to switch 3, a flexible region within the GTPase domain that undergoes conformational changes during Gα _11_ activation (28) (Figs. 1C, 3A and 3E). Arg149 projects into the interdomain interface and is predicted to form 2 contacts (dotted black line) with the switch 3 Asp236 residue (Fig. 3E). Mutation of the Arg149 residue to His149 is predicted to lose both contacts with the Asp236 residue (Fig. 3F).

### Functional characterization of the p.Gly66Ser and p.Arg149His Gα_11_ mutant proteins

**Figure 4.**
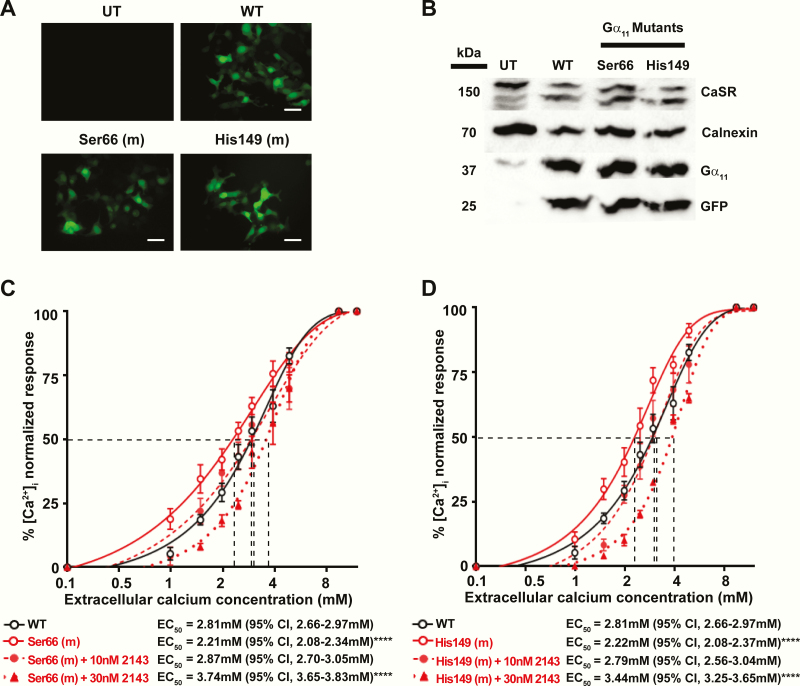
Functional characterization of wild-type (WT) and ADH2-associated mutant Gα _11_ proteins with Gly66Ser and Arg149His substitutions, using intracellular calcium (Ca^2+^_i_) responses, and assessing the effects of the calcilytic NPS 2143 (2143). (A) Fluorescence microscopy of HEK293 cells stably expressing CaSR (HEK-CaSR) and transiently transfected with WT (Gly66 and Arg149) or ADH2-associated mutant (m) Ser66 or His149 pBI-CMV2-*GNA11* constructs. UT, untransfected cells. GFP indicates successful transfection and expression of Gα _11_ by these constructs. Bar indicates 10 μm. (B) Western blot analysis of CaSR, Gα _11_, and GFP using lysates from HEK-CaSR cells transiently transfected with WT or mutant Ser66 or His149 expression constructs. Calnexin was used as a loading control. (C-D) Ca^2+^_i_ response to changes in [Ca^2+^]_e_ of HEK-CaSR cells transfected with: (C) WT or Ser66 Gα _11_ mutant or (D) WT or His149 Gα _11_ mutant, measured by Fluo-4 calcium assays. (C-D) The Ca^2+^_i_ responses to changes in [Ca^2+^]_e_ are expressed as a percentage of the maximum normalized responses and shown as the mean ± SEM of 4–8 independent transfections. The Ser66 and His149 Gα _11_ mutants led to a leftward shift in the concentration-response curve (solid red line). The addition of 10 nM (red dashed line) or 30 nM (red dotted line) 2143 rectified the leftward shift of the Ser66 and His149 Gα _11_ mutants, when compared with WT (black line). The mean half-maximal concentration (EC_50_) of the responses with 95% confidence intervals (CI) and *P* values are shown below for each mutant compared with the WT response. Statistical analysis was performed using the *F-*test. ****P < 0.0001.

The effects of the p.Gly66Ser and p.Arg149His mutations on Gα _11_ function could not be predicted from the homology modeling studies described previously, and we therefore characterized these mutations *in vitro* to determine their effects on CaSR-mediated signaling. HEK-CaSR cells were transiently transfected with pBI-CMV2-*GNA11* constructs expressing either the WT (Gly66 or Arg149) or mutant (Ser66 or His149) Gα _11_ proteins. This bidirectional pBI-CMV2 vector allows for coexpression of Gα _11_ and GFP at equivalent levels (2); and expression of the CaSR, Gα _11_ and GFP was confirmed by fluorescence microscopy and/or Western blot analyses (Fig. 4A, B). Gα _11_ expression was shown to be similar in cells transiently transfected with WT or mutant proteins, and greater in transfected cells than endogenous Gα _11_ protein expression in untransfected cells, by Western blot analyses in which calnexin was used as a loading control (Fig. 4B).

#### Effect of the p.Gly66Ser and p.Arg149His Gα_11_ mutant proteins on CaSR-mediated Ca^2+^_i_ responses.

The effects of the Gα _11_ mutants, Ser66 and His149, on Ca^2+^_e_-induced Ca^2+^_i_ responses using the Fluo-4 calcium assay were assessed, as reported (16). The Ca^2+^_i_ responses in WT and mutant Gα _11_-expressing cells were shown to increase in a dose-dependent manner following stimulation with increasing concentrations of Ca^2+^_e_. The responses of the mutant Ser66 and His149 expressing cells were similar to WT cells at low (0.1 mM) Ca^2+^_e_, but were significantly elevated compared with WT cells following Ca^2+^_e_ stimulation (Fig. 4C, D). Thus, the Ser66 and His149 mutant expressing cells showed a leftward shift in the concentration-response curve (Fig. 4C, D), with significantly reduced mean EC_50_ values (*P* < 0.0001, n = 4–8) of 2.21 mM (95% confidence interval [CI], 2.08-2.34 mM) for Ser66, and of 2.22 mM (95% CI, 2.08-2.37 mM) for His149 expressing cells, compared with 2.81 mM (95% CI, 2.66-2.97 mM) for WT expressing cells (Fig. 4C, D), consistent with a gain-of-function of the Gα _11_ mutants. Addition of 10nM of the negative allosteric modulator NPS 2143 was able to increase the EC_50_ of the mutant cells to a value of 2.87 mM (95% CI, 2.70-3.05 mM) for the Ser66 expressing cells and to 2.79 mM (95% CI, 2.56-3.04 mM) for the His149 cells, such that the responses were not significantly different to WT expressing cells (Fig. 4C, D). Addition of NPS 2143 at the higher 30-nM dose increased the EC_50_ of the Ser66 and His149 mutant cells to values of 3.74 mM (95% CI, 3.65-3.83 mM), and 3.44 mM (95% CI, 3.25-3.65 mM), respectively, which were significantly greater than that of untreated WT cells (*P* < 0.0001, Fig. 4C, D). Therefore, a 10-nM dose of NPS 2143 is effective at normalizing Ser66 and His149 Ca^2+^_i_ responses, whereas 30 mM of NPS 2143 leads to a dose-dependent “overcorrection” that is equivalent to a loss-of-function of the CaSR.

**Figure 5.**
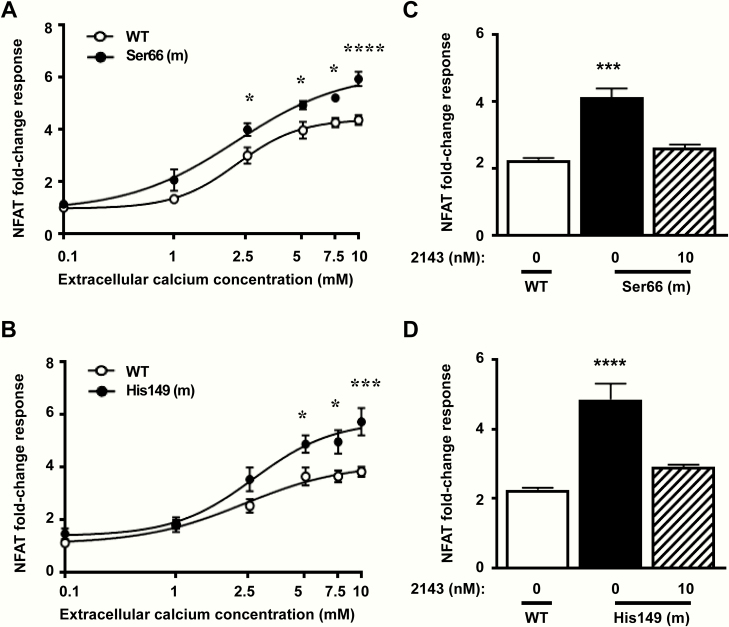
NFAT-response element (NFAT-RE) luciferase reporter responses of the Ser66 and His149 Gα _11_ mutants and effects of NPS 2143 (2143) treatment. [Ca^2+^]_e_-induced NFAT-RE luciferase reporter responses of HEK-CaSR cells transfected with WT (open symbols) or mutant (m) (filled symbols) (A) Ser66 or (B) His149 Gα _11_ proteins. Responses at each [Ca^2+^]_e_ are expressed as a fold-change of low (0.1 mM) [Ca^2+^]_e_ responses, and shown as mean ± SEM of 12 independent transfections. NFAT-RE luciferase reporter activity increased in all cells in a concentration-dependent manner but was significantly greater in mutant cells. Statistical analysis comparing mutant to WT responses by 2-way ANOVA with Tukey’s multiple-comparisons test. (C-D) [Ca^2+^]_e_-induced NFAT-RE reporter responses to 7.5 mM Ca^2+^_e_ in HEK-CaSR cells transfected with WT (open bar) or mutant (filled bar) (C) Ser66 or (D) His149 Gα _11_ proteins. The addition of 10 nM NPS 2143 rectified the elevated NFAT-RE reporter responses of the mutant (hatched bars) to WT (open bars) levels. Responses are expressed as a fold-change of low (0.1 mM) [Ca^2+^]_e_ responses, and shown as mean ± SEM of 4 independent transfections. Statistical analyses were performed by 1-way ANOVA with Dunnett’s multiple comparisons test compared to the WT response. ****P < 0.0001, ***P < 0.001, *P < 0.05 for all panels.

To provide further evidence that the Ser66 and His149 Gα _11_ mutant proteins affect Ca^2+^_i_ signaling, the gene transcription induced by a NFAT-RE containing luciferase reporter construct was measured, as NFAT is a downstream mediator of Ca^2+^_i_ signaling (29) (Fig. 1A). HEK-CaSR cells were transiently transfected with WT or mutant Ser66 or His149 mutant Gα _11_ proteins, and NFAT-RE reporter fold-change responses measured in response to increasing concentrations of Ca^2+^_e_. NFAT-RE reporter responses were significantly elevated in cells expressing the Ser66 and His149 mutant Gα _11_ proteins (Fig. 5A, B). The effects of 10 nM NPS 2143 on these NFAT-RE responses were assessed at 7.5 mM Ca^2+^_e_ concentration. This confirmed the significantly increased NFAT-RE reporter fold-change responses in Ser66 and His149 cells, compared with WT expressing cells (Ser66 = 4.12 ± 0.27 and His149 = 4.85 ± 0.45, compared with 2.26 ± 0.06 for WT expressing cells, *P* < 0.001 and *P* < 0.0001, respectively) and demonstrated that addition of 10 nM NPS 2143 to the cells rectified NFAT-RE reporter fold-change responses to WT values (Ser66 + 10 nM NPS 2143 = 2.63 ± 0.09 and His149 + 10 nM NPS 2143 = 2.92 ± 0.06) (Fig. 5C, D).

#### Effect of the p.Gly66Ser and p.Arg149His Gα_11_ mutant proteins on CaSR-mediated MAPK responses.

**Figure 6.**
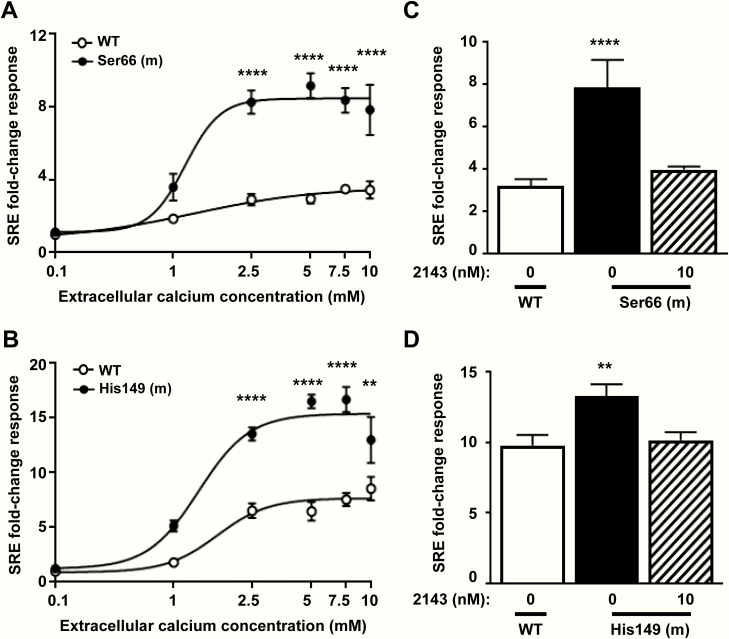
SRE luciferase reporter responses of the Ser66 and His149 Gα _11_ mutants and effects of NPS 2143 (2143) treatment. [Ca^2+^]_e_-induced SRE luciferase reporter responses of HEK-CaSR cells transfected with WT (open symbols) or mutant (m) (filled symbols) (A) Ser66 or (B) His149 Gα _11_ proteins. Responses at each [Ca^2+^]_e_ are expressed as a fold-change of low (0.1 mM) [Ca^2+^]_e_ responses, and shown as mean ± SEM of 8 independent transfections. The Ser66 and His149 Gα _11_ mutants led to significantly increased SRE fold-change responses following stimulation with Ca^2+^_e_ compared with WT Gα _11_. Statistical analysis comparing mutant to WT responses by 2-way ANOVA with Tukey’s multiple-comparisons test. (C-D) [Ca^2+^]_e_-induced SRE reporter responses to 7.5 mM Ca^2+^_e_ in HEK-CaSR cells transfected with WT (open bar) or mutant (filled bars) (C) Ser66 or (D) His149 Gα _11_ proteins. The addition of 10nM NPS 2143 rectified the elevated SRE reporter responses of the mutant (hatched bars) to WT (open bars) levels. Responses are expressed as a fold-change of low (0.1 mM) [Ca^2+^]_e_ responses, and shown as mean ± SEM of 4–8 independent transfections. Statistical analyses were performed by 1-way ANOVA with Dunnett’s multiple comparisons test compared to the WT response. ****P < 0.0001, **P < 0.01 for all panels.

Previous studies of Gα _11_ mutations have demonstrated an increase in MAPK signaling in cells expressing ADH2-causing mutant proteins (9, 17). To investigate the effect of the Ser66 and His149 mutant proteins on MAPK signaling, gene transcription induced by a SRE containing luciferase reporter construct, which is a downstream mediator of MAPK signaling (21) (Fig. 1A), was measured in HEK-CaSR cells transiently expressing WT or mutant Ser66 or His149 Gα _11_ proteins (Fig. 6A, B). Cells expressing the Ser66 and His149 mutant proteins showed no alterations in SRE reporter fold-change responses at low (0.1 mM) Ca^2+^_e_ (Fig. 6A, B). However, stimulation with increasing Ca^2+^_e_ concentrations led to significantly elevated SRE reporter fold-change responses at 2.5 to 10 mM Ca^2+^_e_ in cells expressing the Ser66 and His149 Gα _11_ mutants compared with WT expressing cells (Fig. 6A, B). The effects of 10 nM NPS 2143 on these SRE responses were assessed at 7.5 mM Ca^2+^_e_ concentration. This revealed that the SRE reporter fold-change responses in Ser66 and His149 expressing cells were significantly elevated compared with WT expressing cells (Ser66 = 7.86 ± 1.28, compared with 3.21 ± 0.32 for WT expressing cells, *P* < 0.0001, and His149 = 13.26 ± 0.85, compared with 9.56 ± 1.18 for WT, *P* < 0.01) (Fig. 6C, D), whereas addition of 10 nM NPS-2143 to the Gα _11_ mutant expressing cells rectified SRE reporter responses to that of WT Gα _11_-expressing cells (Ser66 + 10 nM NPS-2143 = 3.96 ± 0.16; and His149 + 10 nM NPS-2143 = 10.15 ± 0.57) (Fig. 6C, D). Thus, a 10-nM dose of NPS-2143 is effective at normalizing mutant Gα _11_ Ser66 and His149 MAPK responses.

## Discussion

Our studies have identified 2 novel heterozygous germline Gα _11_ mutations associated with ADH2. The affected individuals harboring the gain-of-function p.Gly66Ser and p.Arg149His Gα _11_ mutations had a generally mild clinical phenotype with serum calcium concentrations of >1.90 mmol/L and were either asymptomatic or experienced paresthesiae. In addition, there were no alterations in serum concentrations of phosphate or magnesium, and plasma PTH concentrations were detectable and inappropriately within the normal range. These findings are similar to that reported for other patients with ADH2, which is characterized by mild-to-moderate hypocalcemia, normal or elevated serum phosphate, normomagnesemia, and low/normal PTH values (2, 9–12). Urinary calcium excretion was normal or low in the affected individuals in this report, which is also consistent with the phenotype of ADH2. However, some ADH2 patients are susceptible to treatment-related hypercalciuria, nephrocalcinosis, and nephrolithiasis (9, 11, 12).

The p.Gly66Ser and p.Arg149His Gα _11_ mutations reported in this study are located at the interface between the GTPase and helical domains (Figs. 1 and 3). The interdomain interface represents a highly conserved region of the Gα subunit (30), and is the site of multiple interactions between the GTPase and helical domains, including between the linker 1 peptide and the α1 and αA helices (27), and also between the αD-helix and the switch III region (31). This region has a critical structural role within the G-protein and is important for binding guanine nucleotides. In support of this, engineered mutations of the Gα subunit interdomain interface residues have been shown to destabilize the GDP-bound state, and it is likely that such mutations enhance the separation of the GTPase and helical domains, which in turn leads to the release of GDP (30). The interdomain interface region has previously been associated with 4 germline mutations of Gα _11_, three associated with ADH2 (p.Arg60Cys, p.Arg60Leu, and p.Arg181Gln), and 1 associated with FHH2 (p.Thr54Met) (Fig. 1) (2, 9, 10, 15). Additionally, the germline Gα _11_ hypermorphic variant, p.Ile62Val, identified in an N-ethyl-N-nitrosourea generated mouse, which is a model for ADH2, and the somatic constitutively activating mutations in Gα _11_ identified in patients with uveal melanoma, also affect the interdomain interface (18, 32, 33). Thus, this region likely represents a hotspot for disease-causing Gα _11_ mutations.

Our finding that the germline p.Gly66Ser and p.Arg149His Gα _11_ mutations led to a gain-of-function contrasts with engineered mutagenesis studies involving homologous residues in other Gα subunits (27, 31). Thus, an engineered p.Gly66Asp mutation in the closely-related Gα _q_ protein did not cause a gain-of-function, but instead increased coupling of non-G_q_-GPCRs to G_q_ effectors (27, 34). The Gly66 residue is located within the linker 1 peptide, which is not fully conserved between the Gα _11_ and Gα _q_ proteins (Fig. 3A); this lack of sequence conservation may explain the differences observed in these studies. Mutations of the αD-helix Asn167 Gαs residue, which is homologous to the Arg149 Gα _11_ residue, also did not lead to a gain-of-function (31). Indeed, an engineered p.Asn167Ala mutation had no effect on Gαs function, whereas an engineered p.Asn167Arg mutation impaired GPCR-mediated activation of Gαs (31). However, mutagenesis studies of Arg144 in Gα _i_, which is homologous to Arg149 in Gα _11_, did show an increase in GDP dissociation rates, which may increase signaling activity (35). Moreover, the Ser140-Asp227 interdomain contact in Gα _t_, equivalent to Gα _11_ Arg149-Asp236, is important for conformational transitions between active and inactive states (36). Thus, it is difficult to predict the structure-function consequences of the His149 Gα _11_ mutation, and the introduction of the mutant residue, rather than loss of the WT residue in the αD-helix is likely to be responsible for influencing Gα subunit function.

Our *in vitro* studies have shown that that the germline p.Gly66Ser and p.Arg149His Gα _11_ mutations do not enhance CaSR-mediated signaling at low (0.1 mM) Ca^2+^_e_ concentrations, and thus these mutations are not constitutively activating. This observation is in keeping with other reported germline ADH2-causing Gα _11_ mutations, but contrasts with somatic uveal melanoma-causing Gα _11_ mutations, which cause a marked increase in MAPK activation in unstimulated cells (17). The p.Gly66Ser and p.Arg149His Gα _11_ mutations were associated with an overall mild increase in CaSR-mediated Ca^2+^_i_ and MAPK responses, and these findings may explain the mild hypocalcemia observed in the patients harboring these mutations. These cellular studies involving the mutant Ser66 and His149 Gα _11_ proteins have also provided further evidence of the utility of calcilytic compounds in rectifying signaling abnormalities in the Gα _11_ protein, which we have previously shown *in vitro* and in a mouse model of ADH2 (17, 18). Importantly, our studies showed that a low dose (10 nM) of the NPS 2143 calcilytic compound can successfully correct the gain-of-function associated with both the Ser66 and His149 ADH2-causing Gα _11_ mutations, and this is similar to the p.Arg181Gln mutation, which is also located in the interdomain interface (17), but contrasts to the p.Phe341Leu mutation, which affects the α5-helix of the GTPase domain that directly binds to the GPCR transmembrane domains and intracellular loops, and requires a higher dose (30 nM) of NPS 2143 to normalize CaSR signaling (17). Thus, mutations affecting residues in the interdomain interface require a lower dose of allosteric modulator to rectify CaSR signaling than Gα _11_ mutations located in the G-protein-GPCR interface, and further investigation of these may provide insights into the mechanism by which allosteric modulators rectify CaSR-mediated signaling abnormalities associated with G-protein mutations.

In summary, our studies have identified disease-causing mutations located in the linker 1 peptide and αD-helix of the Gα _11_ protein. These findings demonstrate that the Gα _11_ interdomain interface represents a hotspot for germline gain-of-function mutations causing ADH2.

## Abbreviations

ADH: autosomal dominant hypocalcemia
Ca2+e: extracellular calcium
Ca2+i: intracellular calcium
CaSR: calcium-sensing receptor
CI: confidence interval
EC50: mean half-maximal concentration
FHH: familial hypocalciuric hypercalcemia
Gα11: G-protein subunit α11
GPCR: G-protein coupled receptor
NFAT-RE: nuclear factor of activated T-cell response element
pERK: phospho-ERK
PLC: phospholipase C
SFM: serum-free media
SRE: serum response element
WT: wild-type

## Additional Information

***Disclosure Summary:*** The authors have declared that no conflict of interest exists.

***Data availability:*** The datasets generated during and/or analyzed during the current study are not publicly available but are available from the corresponding author on reasonable request.
